# Synthetic Computed Tomography generation using deep-learning for female pelvic radiotherapy planning

**DOI:** 10.1016/j.phro.2025.100719

**Published:** 2025-02-01

**Authors:** Rachael Tulip, Sebastian Andersson, Robert Chuter, Spyros Manolopoulos

**Affiliations:** a Northern Centre for Cancer Care – North Cumbria, Newcastle upon Tyne Hospitals NHS Foundation Trust, Carlisle, Cumbria CA2 7HY, UK; b RaySearch Laboratories, Stockholm, Sweden; c Christie Medical Physics and Engineering (CMPE), The Christie NHS Foundation Trust, Wilmslow Road, Manchester M20 4BX, UK; d Division of Cancer Sciences, School of Medical Sciences, Faculty of Biology, Medicine and Health, University of Manchester, Manchester M13 9PL, UK

**Keywords:** Synthetic CT, MR-only, Dose validation

## Abstract

Synthetic Computed Tomography (sCT) is required to provide electron density information for MR-only radiotherapy. Deep-learning (DL) methods for sCT generation show improved dose congruence over other sCT generation methods (e.g. bulk density). Using 30 female pelvis datasets to train a cycleGAN-inspired DL model, this study found mean dose differences between a deformed planning CT (dCT) and sCT were 0.2 % (D98 %). Three Dimensional Gamma analysis showed a mean of 90.4 % at 1 %/1mm. This study showed accurate sCTs (dose) can be generated from routinely available T2 spin echo sequences without the need for additional specialist sequences.

## 1 Introduction

Magnetic Resonance Imaging (MRI) is increasingly incorporated into radiotherapy planning for treatment of gynaecological tumours [1], [2], [3]. Its advantages include superior soft-tissue visualisation over computed tomography (CT), lack of radiation exposure, and potential incorporation of functional information for adaptive treatment protocols [4], [5], [6], [7], [8].

The lack of a relationship between electron density and MR image intensity requires an MR-CT registration for dose calculation [5]. This registration has inherent uncertainties compounded by anatomical changes between the scans, due to non-simultaneous acquisition [9], [10], [11]. Consequently there is increased interest in generating Synthetic Computed Tomography (sCT) datasets from the MRI scans, negating the need for image registration.

Numerous methods of generating sCT are reported in the literature, mostly limited to local implementations and/or involving specialist MR sequences. Deep Learning (DL) algorithms have demonstrated improvements in dose accuracy over bulk density and atlas based methods [6], [7], [12], [14], [15], [16], [17]. By removing the need for specialist sequences such as Dixon or ultra-short echo, DL solutions based sCT generation utilising T2 spin echo sequences have the advantages of reducing the patient appointment duration, facilitating introduction of specialised treatments and improving confidence in longer term sustainability of the sCT solution [13], [14], [18].

Most pelvis studies for sCT generation have focussed on male anatomy [19], [20], [21]. O’Connor *et al*, suggested a single DL model for sCT generation could be used for both male and female patients [22]. Whilst in dose terms this has little impact, their study did not consider the impact of using a male DL model on a female patient and vice versa. Additionally, sCT is now being incorporated into the radiotherapy workflow for treatment verification where anatomical differences will have increased significance [23], [24], [25].

This study reports the results of a DL model being developed by a commercial company for sCT generation of female pelvic sites based on a standard T2 spin echo sequences.

## 2 Materials and methods

### 2.1 Image acquisition and model training

Thirty female pelvis MRI datasets, were used to train the model for sCT generation (28 training, 2 validation). The sample size was determined using the findings of Boulanger *et al*, [26]. External markers (e.g. ball bearings) and patient support systems were removed.

All MRI data was acquired on a Siemens Espree (Siemens Healthineers, Germany) 1.5 T MRI scanner using a 3D T2 TSE sequence with full 3D distortion correction (voxel size 0.141 × 0.141 × 0.15 cm). All CT data was acquired on a Siemens Confidence (Siemens Healthineers, Germany) 64 slice CT scanner 120 kV (voxel size 0.107 × 0.107 × 0.25 cm). The median time between data acquisitions was 18 h 43 min (range: 15 min to 138 h 35 min) where half the time, the CT was acquired prior to the MRI. Scans were acquired in the treatment position on an indexed flat couch with knee rest for patient position stability and reproducibility.

Each MRI and corresponding CT data set were imported into RayStation (RaySearch Laboratories, Stockholm) (V11A Research version).

The methodology described by Zhu *et al*, was used to create the sCT model [13]. The MR-CT pairs underwent an initial deformable registration whose accuracy was assessed via DICE Similarity assessment (DSC) and mean distance to agreement (MDA). The model was trained in a semi-supervised fashion, using a convolutional neural network architecture similar to CycleGAN, but including an extra paired term to account for the existence of paired data in this context. As in CycleGAN, the model consists of two generators, which convert the images from MR to CT (G_CT_) and from CT to MR (G_MR_), and two discriminators which try to discriminate between real and fake images. In the end, it is G_CT_ that will be used in this study.

### 2.2 The test data set

Ten female pelvis datasets (independent of those used for model generation) were used to test the sCT algorithm. This testing sample size is consistent with other published work involving this patient cohort [27], [28], [19], [29]. The MRI and CT scans were acquired on the same scanners with the same patient positioning described previously. The sCT used for testing was derived solely from the patient MR dataset (sCT generation time of 1 min) and compared to a deformed planning CT (dCT) derived from a deformable registration (hybrid intensity – ANACONDA method) of the planning CT (pCT) and MR [30]. This removed the majority of patient position differences seen between the datasets. The dCT represents the same geometry as the MRI, forming the ‘ground truth’ for comparisons to the sCT for dose analysis.

### 2.3 Planning

**Fig. 1:**
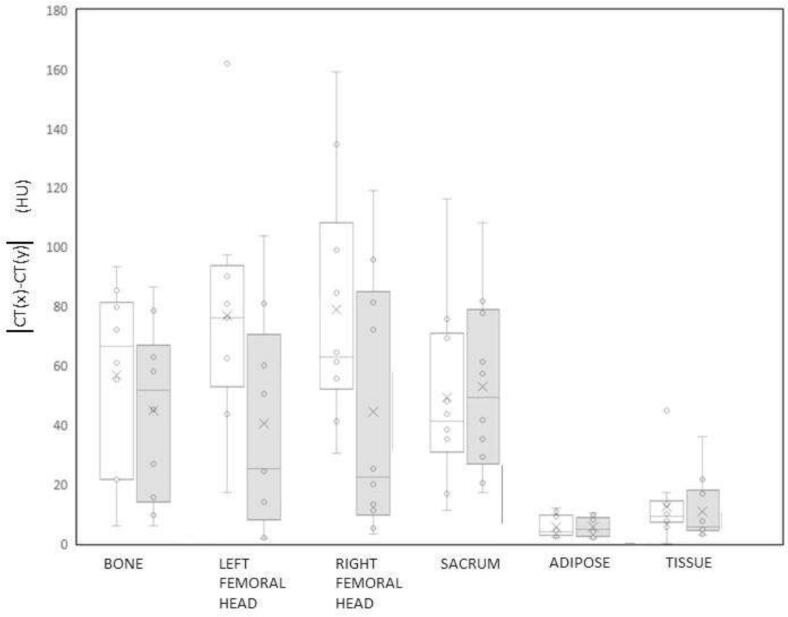
A box and whisker plot showing the absolute error (modulus) for the CT number for sCT vs pCT(non shaded boxes) and sCT vs dCT (shaded boxes).

Contours (Fig. 1) were drawn using MVision AI segmentation (MVision AI, Helsinki, Finland). Tissue and Adipose were contoured using the RayStation V2023b thresholding tool (RaySearch Laboratories, Sweden). Plans consisted of a dual-arc co-optimised arrangement, at 6 MV using the Collapsed Cone algorithm. A standard prescription of 45 Gy in 25 fractions median dose (D50%) to PTV was used (dose grid size 2 mm). The CTV comprised the cervix and uterine structures without nodal chains due to limitations in the length of the MRI images that could be acquired in a representative scan time. The PTV was generated from the CTV using our clinical margins. An initial plan was generated on the pCT with full density heterogeneity applied and then recomputed on the same CT dataset segmented into three structures (pCT_bulk_) air, bone and tissue with bulk densities of 0.001 g/cc and 1.000 g/cc for the air and tissue respectively. Mean bone densities derived from the individual patients were used to overcome variations deriving from effects such as menopausal changes [19], [28], [31], [32].

The bulk density process was repeated for the dCT. A plan (previously described) was generated on the dCT, and recalculated on the sCT.

For each test plan, the sCT were compared with the dCT and pCT in terms of the CT numbers (HU) and MAE, calculated (Eq. (1) for the three combinations: sCT and pCT, pCT and dCT and between sCT and dCT where N is the number of patients.

(1) $$\mathrm{MAE}=\frac{1}{N}\sum_{i=1}^{N}\left|{\mathrm{sCT}}_{i}-{\mathrm{pCT}}_{i}\right|$$

MAE were calculated for bones, femoral heads, sacrum, adipose and tissue. Following QQ testing of the paired differences for distribution normality, dose volume histogram parameters were compared using a paired Student *t* test (p = 0.05) for significance.

The plans calculated on the dCT were compared to the same plans calculated on the sCT, using 3D gamma analysis matrices (MICE toolkit 1.1.3, NONPI Medical AB, Sweden) at 3 %/3 mm, 3 %/2 mm, 2 %/2 mm and 1 %/1 mm with a low dose threshold of 20 %.

## 3 Results

DSC and MDA between the training datasets showed values of 0.97±0.04 and 0.2 cm±0.05 cm respectively for the body. For pelvic bones and femoral heads, the DSC was 0.82 ±0.1 and 0.86 ± 0.1 respectively. MDA for pelvic bones and femoral heads was 0.2 cm ± 0.1 cm.

Analysis of the sCT found mean CT numbers for bone, femoral heads and sacrum were lower on the sCT compared with the pCT (bone 333 HU_sCT_ ±27 HU_sCT_ vs 373.1 HU_pCT_ ±51 HU_pCT_, femoral heads 278 HU_sCT_ ±32 HU_sCT_ vs 351 HU_pCT_ ±48 HU_pCT_ and sacrum 190 HU_sCT_±44 HU_sCT_ vs 225 HU_pCT_ ±49 HU_pCT_). Bone density in the original training datasets was comparable with that in the test datasets (mean 1.34 g/cm^3^ ±0.04 g/cm^3^ compared with 1.36 g/cm^3^ ±0.13 g/cm^3^ respectively) therefore differences in density/HU are attributed to the sCT generation. CT numbers for adipose and soft tissue were comparable (adipose −95 HU_sCT_ ±6 HU_sCT_ vs −99 HU_pCT_ ±10 HU_pCT_, soft tissue 30 HU_sCT_ ±13 HU_sCT_ vs 31 HU_pCT_ ±19 HU_pCT_) (Fig. 1).

**Fig. 2:**
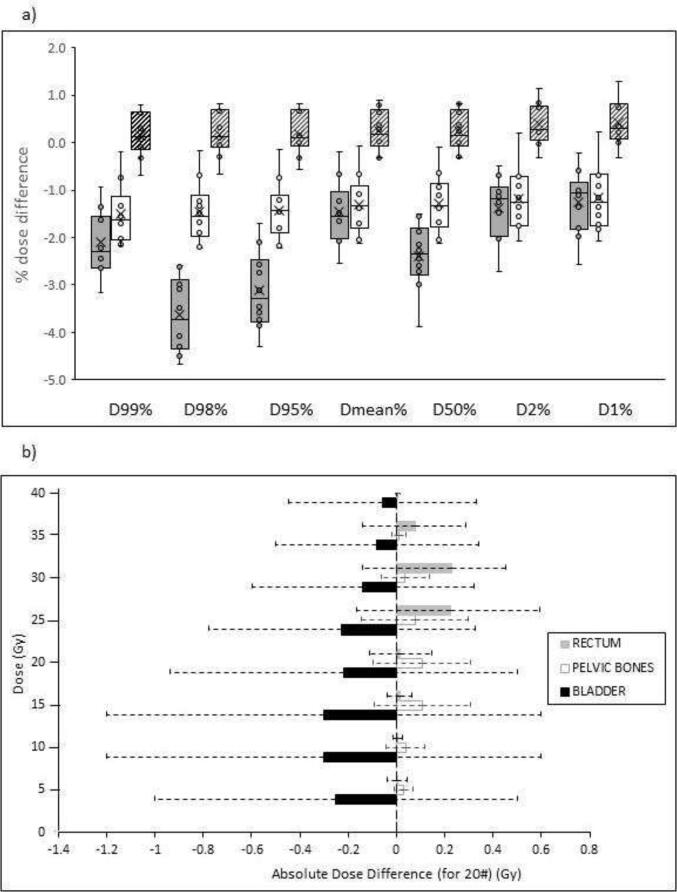
A) the ptv dose differences with bulk assignment for the pCT (solid filled boxes) and dCT (unshaded boxes). The PTV dose difference, with full CT heterogeneity applied, between dCT and sCT is shown in the crossed boxes. b) The mean absolute dose difference for the OARs between the dCT and the sCT with full CT heterogeneity applied. The error bars represent 1 standard deviation.

The mean dose difference for D98% between the pCT and pCT_bulk_ was −3.6 % (SD 0.8 %). For the DL method of sCT generation, the mean dose difference between the doses calculated using dCT and sCT for D98% was reduced to 0.2 % (SD 0.5 %) (Fig. 2a). Student 't' testing of the doses between dCT and sCT (p = 0.05) found the differences were significant for D2% and D1%.

For the OARs, the mean dose differences between the dCT and sCT were less than 2 % (Fig. 2b). The largest dose differences were seen around the external contour (∼3%). These were attributed to residual anatomical variations arising from the deformable registration between the dCT and the sCT.

Gamma analysis showed mean values for 3 %/3 mm, 3 %/2 mm, 2 %/2 mm and 1 %/1 mm of 99.4 %, 99.2 %, 97.1 % and 90.4 % respectively. Patient 10 showed the worse results with gamma values of 98.6 %, 98.2 %, 94.1 % and 79.8 % for 3 % /3 mm, 3 %/2 mm, 2 %/2 mm and 1 %/1 mm respectively.

## 4 Discussion

This is one of the first studies that assesses sCT dose accuracy for gynaecological cancer treatments using a commercially developed cycleGAN DL algorithm.

Our model was trained using standard T2 TSE sequences reducing the risk of artefacts arising from anatomical changes such as bladder filling and improving confidence in the longer term sustainability of the sCT solution [15], [18], [33], [34], [35], [36].

Bony structures were the main areas of CT number discrepancy between the pCT, sCT and dCT attributed, in part, to the voxel resampling that occurs during the deformable registration process part of the model training process. This was also reported by Farjam *et al* [7] and Kraus *et al* [38]. Our value of average MAE of 46.7 HU is comparable to reported MAE values ranging from 36.5±4.1 HU to 74.3 ±10.9 HU) [37], [38], [39], [40], [41], [42], [43]. The larger difference in CT number between dCT and pCT compared with dCT and sCT is attributed to anatomical changes that occur between the planning CT scan and the MR scan and the change in voxel size between these 2 imaging modalities that derives from the deformable registration required to create the dCT.

For the sCT and dCT, the mean dose difference for D98 was 0.2 %±0.5 % and mean D2 was 0.4 %±0.5 %. The GAN method by Maspero *et al*, reported that doses to the target were in general around 0.1 to 0.3 % with maximum difference of 1.6 %. This study found the maximum difference to the target was 1.3 % [33]. The sCT doses were generally higher than those in the dCT. Comparison to studies where DL was not used, found dose differences for target volumes ranged from 0 % to 9.7 % where the largest differences were reported for bulk density assignment [37], [42], [43], [44]. A study limitation is that the dose comparison between the sCT and dCT is not perfect due to registration uncertainties from the derivation of dCT from the pCT [45].

Global 3D gamma analysis for dCT vs sCT found a mean value of 97.0 % (SD 1.6 %) for 2 %/2mm and 90.4 % (SD 5.8 %) for 1 %/1mm. Maspero et *al* [33] reported gamma pass rates of 91 % for 2 %/2mm whereas Bird et al [46] reported gamma values greater than 99 % at 1 %/1mm for their ano-rectal study using cGAN whereas ours were all below 98 %. They had 46 patients for their training data compared with our 30 patients indicating our results would be improved by increasing the training MR-CT pairs. For one patient, the gamma pass values were poorer which was attributed to the patient’s larger anatomical size. The additional tissue mobility resulted in shape differences between the dCT and the sCT. Additionally, the PTV extends superiorly to the superior edge of the sCT where accuracy is limited by the original MRI scan length. Incorporating a representative number of bariatric patients into model training should be considered when implementing an MR-only pathway.

The study did not include patients with metal implants in the test or training data. Whilst the MR and CT scanners have metal artefact reduction software, the exclusion of these patients was deliberate to avoid uncertainties deriving from image artefacts (such as those from susceptibility (MR) or electron density starvation (CT)). Koivula *et al*, showed promising sCT generation results involving Dixon based methods [47]. Future work would involve consideration of the effect of metal implants on the DL algorithm.

To improve the results for other sequences and MRI machines, the training set should be expanded to include the additional sequences and machines [48], [49]. Our version of the algorithm was a prototype. It is envisaged a commercial version would not require training on center specific MRIs. Studies have been reported, with promising results, incorporating training datasets from multiple centers [46], [49]. This requires consideration of validation processes amongst the scientific community to enable the future development of this technology [18], [50].

This study showed accurate sCT datasets (dose) can be generated from routine T2 TSE sequences using a commercially developed cycleGAN-inspired DL algorithm. Benefits include reducing the patient appointment duration and improved confidence in the longer term sustainability of the sCT solution.

## Declaration of competing interest

The authors declare the following financial interests/personal relationships which may be considered as potential competing interests: The authors have a research agreement with RaySearch Laboratories from which they received technical support for the sCT generation algorithm. S Andersson is an employee of RayStation Laboratories and developed the deep learning algorithm and assisted with generating the female pelvis model. However, RaySearch Laboratories had no part in the design or execution of the validation aspects of the study.
